# Vitrectomy and All-Cause and Cause-Specific Mortality in Elderly Patients With Vitreoretinal Diseases: A Nationwide Cohort Study

**DOI:** 10.3389/fmed.2022.851536

**Published:** 2022-04-25

**Authors:** Yoon Jeon Kim, Ji Sung Lee, Yunhan Lee, Hun Lee, Jae Yong Kim, Hungwon Tchah

**Affiliations:** ^1^Department of Ophthalmology, Asan Medical Center, University of Ulsan College of Medicine, Seoul, South Korea; ^2^Clinical Research Center, Asan Institute for Life Sciences, Asan Medical Center, University of Ulsan College of Medicine, Seoul, South Korea; ^3^Department of Clinical Epidemiology and Biostatistics, Asan Medical Center, University of Ulsan College of Medicine, Seoul, South Korea

**Keywords:** vitrectomy, nationwide cohort study, cause specific mortality, all-cause mortality, vitreoretinal disease

## Abstract

**Purpose:**

To determine the all-cause and cause-specific mortality in elderly patients with vitreoretinal diseases based on vitrectomy status.

**Methods:**

Elderly patients (aged ≥ 60 years) diagnosed with vitreoretinal diseases between 2003 and 2012 using the Korean National Health Insurance Service-Senior cohort (2002–2015) were included in this nationwide population-based retrospective cohort study. The exposure of interest was vitrectomy, and information on mortality from patient inclusion until December 2015 was obtained. Cox regression modeling was used to assess the association between vitrectomy and mortality. An additional subgroup analysis was performed to investigate the effects of the underlying retinal disease characteristics and comorbidities on mortality.

**Results:**

The study cohort included 152,283 patients (3,313 and 148,970 in the vitrectomy and non-vitrectomy groups, respectively). The adjusted model showed vitrectomy was associated with a decreased risk of pulmonary-cause mortality [hazard ratio (HR), 0.51; *P* < 0.001]; however, no association was observed for all-cause mortality (HR, 0.93; *P* = 0.325). Vitrectomy was associated with increased mortality risk (all-cause: HR, 1.26; *P* < 0.001 and vascular causes: HR, 1.41; *P* = 0.003) among patients with retinal vascular diseases and decreased mortality risk (all-cause: HR, 0.64; *P* < 0.001 and pulmonary causes: HR, 0.35; *P* = 0.011) among patients with macular diseases. There were significant interactions between age and vitrectomy with respect to all-cause mortality among patients with either vitreoretinal disease.

**Conclusions:**

In elderly patients with retinal diseases, the vitrectomy group showed the lower mortality from pulmonary causes with no association for all-cause mortality.

## Introduction

In the last few decades, the clinical efficacy and safety of vitrectomy have significantly improved owing to advancements in surgical instruments and equipment (1, 2). In addition, patient discomfort during the postoperative period has also decreased. Accordingly, while vitrectomy was initially only performed for severe cases, such as retinal detachment with large retinal breaks or proliferative vitreous retinopathy, the indications for surgery have been expanding (3). An increasing number of patients worldwide are undergoing surgery, particularly the elderly (4). Therefore, it is worthwhile to analyze whether there is a difference in mortality rates depending on the performance of vitrectomy in the elderly population.

Various previous studies have evaluated the association between ocular surgeries and long-term mortality, particularly between cataract surgery and mortality (5–7). Additionally, some cohort studies have evaluated the survival rates after vitrectomy in patients with proliferative diabetic retinopathy, reporting a 5-year survival rate between 68 and 96% (8, 9) and a 10-year survival rate of 49% (10). However, those studies in relation to vitrectomy included small sample sizes of people from a single group and no comparisons with a non-surgical group. To the best of our knowledge, no previous study has compared mortality rates according to vitrectomy status, particularly in a population-based cohort. Hence, we aimed to evaluate and compare the all-cause and cause-specific mortality of the elderly Korean population based on vitrectomy status. We used data from the Korean National Health Insurance Service Senior cohort (NHIS-Senior) database, which is a nationwide database that covers the entire older-adult Korean population (11). In addition, a subgroup analysis was performed to determine whether the mortality rate differed according to the two main subtypes of vitreoretinal disease (retinal vascular and macular diseases).

## Methods

### Study Setting

For this population-based retrospective cohort study, data was obtained from the Korean NHIS-Senior database (2002–2015). As previously described, the Korean NHIS is a national health insurance database that includes all patient data related to healthcare and long-term care services (11). The senior cohort data covers 558,147 individuals randomly sampled from 10% of the approximate 5.5 million Koreans aged ≥ 60 years. All participants included in the NHIS-Senior database were followed until 2015 unless they were disqualified for health coverage. The NHIS-Senior database comprises patient data, including age, sex, national health screening, healthcare utilization, disease diagnoses, vitrectomy status, procedures, and prescribed medications, as well as mortality-related information. The patients' healthcare records were not duplicated because all Korean residents receive a unique identification number at birth. The Korean NHIS uses the Korean Standard Classification of Diseases, Seventh Edition (KCD-7) codes and the Korean Electronic Data Interchange (KEDI) codes (12). This database can be used for national healthcare evidence-based analyses that accurately represent the entire elderly population in Korea.

All data in the NHIS-Senior database were de-identified and encrypted to protect the privacy of the participants before use. As the NHIS-Senior database comprises data that are open to the public, the Institutional Review Board (IRB) of the Asan Medical Center waived the requirement for a review of this study (AMC IRB No. 2019-1630).

### Study Population

**Figure 1:**
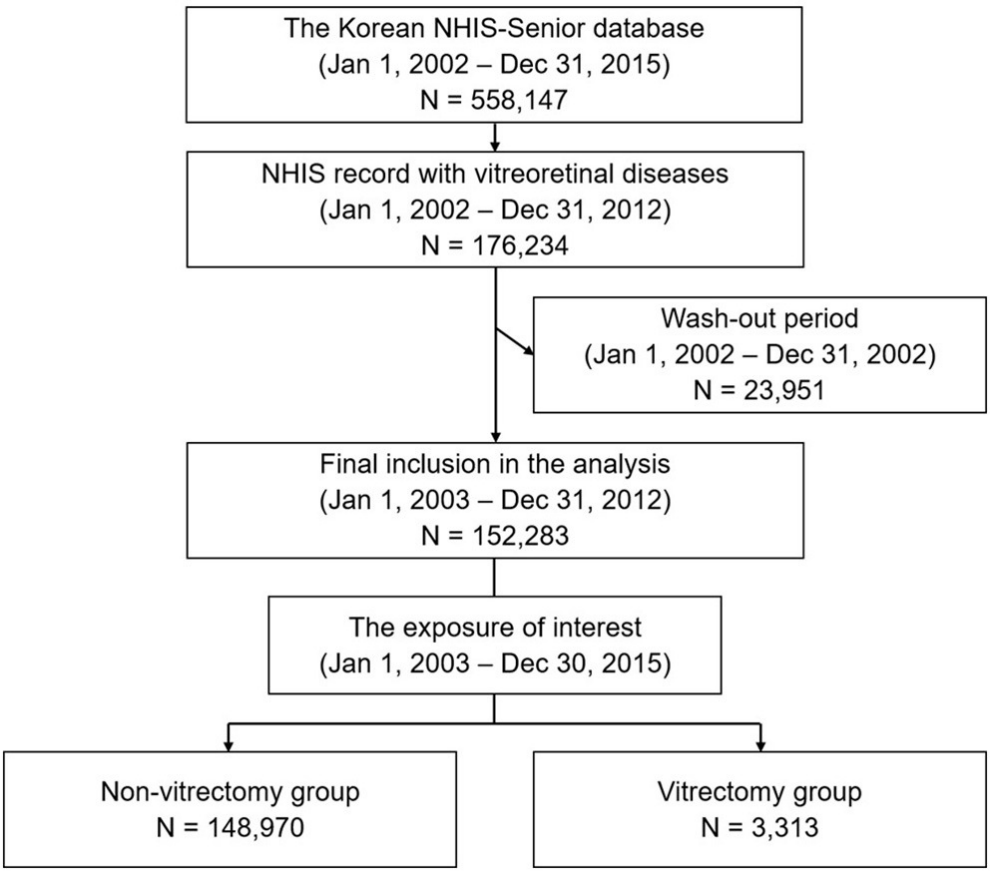
Flow-chart illustrating the study design.

Our target population was Korean older adults who were included in the NHIS-Senior database between January 1, 2002 and December 31, 2015 (*n* = 558,147) (Figure 1). The inclusion criteria were as follows: aged ≥ 60 years, with at least one NHIS record between January 1, 2002 and December 31, 2012 (*n* = 176,234) with a KCD-7 code for a vitreoretinal disease (Supplementary Table 1). In addition, we included a wash-out period between January 1, 2002 and December 31, 2002 to reduce the potential impact of surveillance bias (*n* = 152,283). Patients with the following characteristics were excluded: those with KCD-7 codes for congenital or hereditary vitreoretinal diseases, a ruptured globe, intraocular foreign body, or other retinal/choroidal malignancy.

### Exposure

The exposure of interest was a vitrectomy. The eligible subjects were classified into two groups based on whether they underwent a vitrectomy between January 1, 2003 and December 30, 2015 (Figure 1). The vitrectomy group comprised all participants with the KEDI code for vitrectomy. Vitrectomy was defined as a total vitrectomy (KEDI code S5121). The non-vitrectomy group was the unexposed group, and comprised all participants with diagnostic codes for vitreoretinal diseases, but with no KEDI code for vitrectomy. The patients in both groups were followed up starting at the earliest date the diagnostic code for vitreoretinal diseases was assigned.

### Outcomes

The primary outcomes of interest were all-cause and cause-specific mortality for the period from patient inclusion to December 31, 2015. Mortality was determined based on an indicator variable in the NHIS-Senior database, which contains information on all-cause and cause-specific mortality. In this study, the causes of death were grouped as cancer, accident-related, vascular, pulmonary, neurologic, infectious, or trauma-related deaths (Supplementary Table 2).

We performed a time-dependent analysis to prevent an immortal time bias (13). For the non-vitrectomy group, the time-to-death was calculated as the number of days from the vitreoretinal disease diagnosis to death. For the vitrectomy group, the period between the diagnosis and vitrectomy was considered the follow-up period for vitreoretinal diseases, and the period after surgery was considered the follow-up period for vitrectomy. Therefore, the person-days of follow up before the surgery were classified as the non-surgery group until the surgery day definition was met, and as the surgery group thereafter. Participants who did not have a record of death were followed up until the last known date or December 31, 2015.

### Covariates

The collected demographic data included patients' age at the time of vitreoretinal disease diagnosis, sex, area of residence, and income level. The areas of residence were grouped as either metropolitan or provincial areas according to the administrative unit of Korea. Household income was categorized as below or above the twentieth percentile.

Both systemic and ocular comorbidities were included as covariates in this study. The systemic comorbidities were assessed at the time of vitreoretinal disease diagnosis. The Charlson comorbidity index (CCI) score was used as a covariate to represent patients' overall systemic health (14). The CCI is a weighted index of systemic disease burden based on the presence or absence of 17 systemic comorbidities, with a higher CCI score indicating a higher burden of systemic disease. Based on their systemic disease profiles, patients were assigned a CCI score between 0 and 6 that could be used to predict the 1-year mortality risk (6). In addition, data from a self-reported questionnaire (smoking status, alcohol consumption, and regular exercise) was obtained for a subset of patients who underwent a national health screening program, medical check-ups provided every 2 years.

Other ocular comorbidities, including glaucoma (KCD-7 codes H40 and H42) and severe cataract (KCD-7 codes H25.2 and H25.1), were examined. Since objective visual acuity data were not available, patients with diagnostic codes for Morgagnian-type senile cataract and senile nuclear cataract were considered to have severe cataract subtypes. Determining the presence of systemic and ocular comorbidities was based on the availability of KCD-7 codes for these conditions (Supplementary Table 3).

### Statistical Analysis

The primary objective of this study was to examine the differences in all-cause and cause-specific mortality among patients diagnosed with vitreoretinal diseases between the vitrectomy and non-vitrectomy groups. We used absolute standardized differences (ASD) to compare the baseline characteristics (15). ASD, calculated as differences in the means or proportions divided by a pooled estimate of the standard deviation (SD), is not as sensitive to sample size when compared with the traditional significance testing; hence, it is useful for identifying clinically meaningful differences. An ASD > 0.1 is considered clinically meaningful (16). Cox regression models based on the time-varying covariate vitrectomy status (exposure) were used to determine the covariate-adjusted associations between vitrectomy and time to death from any cause or death attributed to cancer and vascular, pulmonary, neurologic, infectious, or traumatic conditions. We used two models with adjustments for potential confounding factors at baseline. Model 1 was adjusted for age and sex. Model 2 was further adjusted for systemic disease burden as measured using income, area of residence, CCI, and ocular comorbidities. In addition, the adjusted HR of each covariate was used to investigate the effects of age, sex, area of residence, income level, CCI score, glaucoma, and severe cataracts on vitrectomy-related overall mortality. Moreover, subgroup analyses were performed to investigate differences in mortality according to the underlying retinal disease type. The Statistical Analysis System (SAS) program version 9.4 (SAS Institute, Cary, NC, United States) was used for all statistical analyses, and statistical significance was set to a two-sided *p* < 0.05.

## Results

### Baseline Characteristics

The study cohort included 152,283 patients (mean age, 72.3 ± 6.2 years), with 3,313 and 148,970 in the vitrectomy and non-vitrectomy groups, respectively. The baseline characteristics of the study cohort are summarized in Supplementary Table 4. Most of the patients in both groups were diagnosed with vitreoretinal diseases when aged <70 years. The older population (≥ 75 years) were not likely to undergo vitrectomy (ASD = 0.4047). More patients who underwent vitrectomy lived in metropolitan areas than in provincial areas (ASD = 0.1085) and had higher incomes (ASD = 0.1214). The remaining demographic factors, including the CCI, were similar between the groups (all ASD <0.1). A greater proportion of patients had glaucoma in the vitrectomy group (49.4 and 38.5% in the vitrectomy and non-vitrectomy groups, respectively; ASD = 0.2403). The fasting plasma glucose concentration was higher in the vitrectomy group (ASD = 0.1170), but other parameters were not different between the groups (all ASD > 0.1).

### Mortality Rate

**Figure 2:**
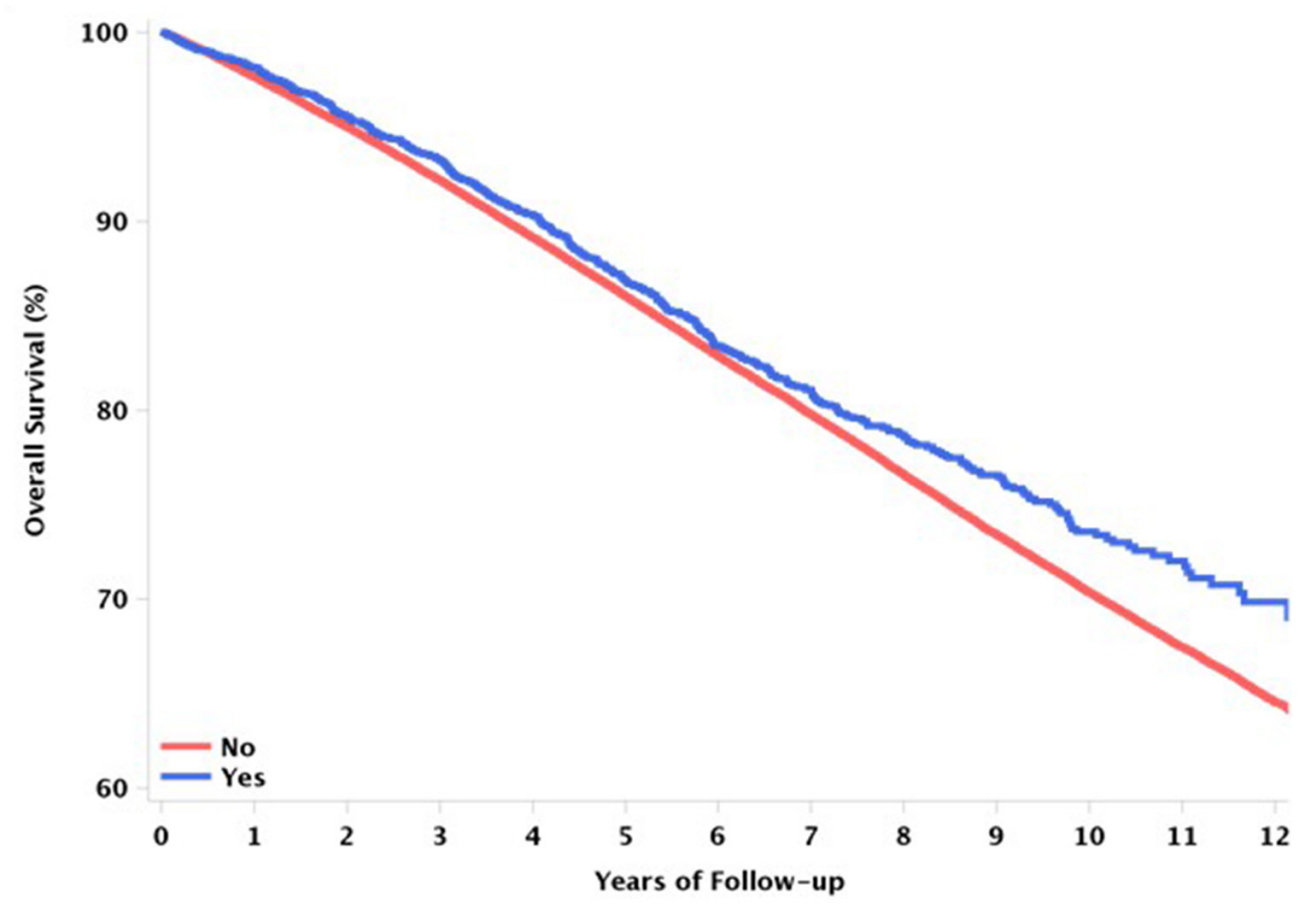
Kaplan–Meier graphs to describe all-cause mortality between the vitrectomy and non-vitrectomy groups.

Table 1 shows the mortality rates of the elderly patients with vitreoretinal diseases stratified by vitrectomy status. The crude mortality at any time during the study period was 2.89 deaths per 100 person-years in the vitrectomy group and 3.36 deaths per 100 person-years in the non-vitrectomy group. The unadjusted model showed a significant difference in the hazard ratios (HRs) for all-cause and cause-specific mortality between the groups (Table 2). Vitrectomy was associated with a lower risk of all-cause mortality [HR, 0.81; 95% confidence interval (CI), 0.74–0.88, *P* < 0.001] and mortality from malignant causes (HR, 0.83; 95% CI, 0.70–0.97, *P* = 0.023), pulmonary causes (HR, 0.40; 95% CI, 0.27–0.58, *P* < 0.001), neurologic causes (HR, 0.46; 95% CI, 0.25–0.86, *P* = 0.014), and infectious causes (HR, 0.44; 95% CI, 0.22–0.88, *P* = 0.021). After adjusting for other variables, the decreased risk of mortality from pulmonary causes present in the vitrectomy group persisted in Model 1 (HR, 0.50; 95% CI, 0.34–0.73*, P* < 0.001) and Model 2 (HR, 0.51; 95% CI, 0.35–0.74*, P* < 0.001). However, the adjusted model showed no association between vitrectomy and all-cause and other cause-specific mortality (*P* > 0.05). Based on this analysis, Kaplan–Meier survival curves were calculated to describe all-cause mortality according to the vitrectomy status (Figure 2).

**Table 1:** Mortality rates of elderly patients with vitreoretinal disease stratified by vitrectomy status.

	**Mortality rate, No. of deaths (incidence per 100 person-years; 95% CI)**	
**Cause of mortality**	**Vitrectomy group**	**Non-vitrectomy group**
All-cause	538 (2.89; 2.66–3.15)	36,627 (3.36; 3.33–3.40)
Cancer	143 (0.77; 0.65–0.91)	9,903 (0.91; 0.89–0.93)
Vascular	143 (0.77; 0.65–0.91)	9,008 (0.83; 0.81–0.84)
Pulmonary	27 (0.15; 0.10–0.21)	3,577 (0.33; 0.32–0.34)
Neurologic	10 (0.05; 0.03–0.10)	1,057 (0.10;0.09–0.10)
Infectious	8 (0.04; 0.02–0.09)	980 (0.09; 0.08–0.10)
Accident or trauma	37 (0.20; 0.14–0.28)	2,366 (0.22; 0.21–0.23)

*CI, confidence interval*.

*Total person-years were 18,589 for the vitrectomy group and 1,090,111 for the non-vitrectomy group*.

*Poisson regression model with vitreoretinal vitrectomy status as the time-varying covariate*.

**Table 2:** Hazard ratios for all-cause and cause-specific mortality in elderly patients with vitreoretinal disease stratified by vitrectomy status.

**Cause of mortality**	**Unadjusted Cox model ^a^**		**Adjusted Cox model 1 ^a,b^**		**Adjusted Cox model 2 ^a,c^**	
**(No. of participants)**	**Hazard ratio (95% CI)**	***P*** **-value**	**Hazard ratio (95% CI)**	***P*** **-value**	**Hazard ratio (95% CI)^**b, c**^**	***P*** **-value**
All-cause	0.81 (0.74–0.88)	<0.001	0.94 (0.86–1.02)	0.142	0.96 (0.88–1.04)	0.325
Cancer	0.83 (0.70–0.97)	0.023	0.87 (0.74–1.03)	0.103	0.88 (0.75–1.04)	0.143
Vascular	0.86 (0.73–1.02)	0.083	1.04 (0.88–1.23)	0.623	1.07 (0.91–1.26)	0.422
Pulmonary	0.40 (0.27–0.58)	<0.001	0.50 (0.34–0.73)	<0.001	0.51 (0.35–0.74)	<0.001
Neurologic	0.46 (0.25–0.86)	0.014	0.58 (0.31–1.08)	0.088	0.58 (0.31–1.08)	0.088
Infectious	0.44 (0.22–0.88)	0.021	0.53 (0.26–1.06)	0.075	0.53 (0.26–1.06)	0.074
Accident or trauma	0.89 (0.65–1.24)	0.496	0.97 (0.70–1.34)	0.849	0.98 (0.71–1.36)	0.909

*CI, confidence interval*.

a *Cox model with vitreoretinal vitrectomy status as the time-varying covariate*.

b *Adjusted for age and sex*.

c *Adjusted for age, sex, income, area of residence, Charlson comorbidity index (0, 1, 2, 3, 4, ≥ 5), glaucoma, and cataract severity*.

To assess the factors affecting these differences in all-cause mortality between the groups, fully adjusted models were used. A significant interaction between age and vitrectomy-related mortality was found (*P* < 0.001) (Supplementary Table 5). The HR in the vitrectomy group was higher for patients <70 years of age; however, in the vitrectomy group, all-cause mortality decreased in patients aged ≥ 75 years. The lowest mortality in the vitrectomy group was observed for patients aged ≥ 85 years, with a 56% lower HR than that in the non-vitrectomy group (HR, 0.44; 95% CI, 0.25–0.78*, P* = 0.005). The other factors were not associated with mortality rates stratified by vitrectomy status.

### Subgroup Analysis

Subgroup analyses were performed based on the etiology of the retinal disease (i.e., retinal vascular or macular diseases) (Supplementary Tables 6, 7). For retinal vascular diseases (Table 3), the fully adjusted model showed that vitrectomy was associated with a higher risk of mortality from all causes (HR, 1.26; 95% CI, 1.11–1.42, *P* < 0.001) and vascular causes (HR, 1.41; 95% CI, 1.12–1.78, *P* = 0.021). Conversely, for macular diseases (Table 4), the fully unadjusted model showed that vitrectomy was associated with a lower risk of mortality from all causes (HR, 0.64; 95% CI, 0.53–0.78, *P* < 0.001), vascular causes (HR, 0.69; 95% CI, 0.48–1.00, *P* = 0.052), and pulmonary causes (HR, 0.35; 95% CI, 0.16–1.79, *P* = 0.011). In both subgroups, considerable interactions between age and vitrectomy-related mortalities were found (Supplementary Tables 8, 9).

**Table 3:** Hazard ratios for all-cause and cause-specific mortality in elderly patients with retinal vascular diseases stratified by vitrectomy status.

**Cause of mortality**	**Unadjusted Cox model ^a^**		**Adjusted Cox model 1 ^a,b^**		**Adjusted Cox model 2 ^a,c^**	
**(No. of participants)**	**Hazard ratio (95% CI)^**a**^**	***P*** **-value**	**Hazard ratio (95% CI)**	***P*** **-value**	**Hazard ratio (95% CI)**	***P*** **-value**
All-cause	1.04 (0.92–1.16)	0.561	1.19 (1.06–1.34)	0.004	1.26 (1.11–1.42)	<0.001
Cancer	0.90 (0.69–1.16)	0.408	0.94 (0.72–1.22)	0.624	0.99 (0.76–1.29)	0.960
Vascular	1.12 (0.89–1.41)	0.317	1.35 (1.07–1.69)	0.010	1.41 (1.12–1.78)	0.003
Pulmonary	0.60 (0.36–1.00)	0.052	0.75 (0.45–1.26)	0.278	0.78 (0.47–1.31)	0.356
Neurologic	0.37 (0.12–1.17)	0.090	0.48 (0.15–1.49)	0.204	0.48 (0.15–1.51)	0.209
Infectious	0.54 (0.20–1.46)	0.227	0.65 (0.24–1.75)	0.398	0.68 (0.25–1.82)	0.439
Accident or trauma	0.79 (0.45–1.39)	0.413	0.84 (0.48–1.48)	0.547	0.88 (0.50–1.55)	0.653

*CI, confidence interval*.

a *Cox model with vitreoretinal vitrectomy status as the time-varying covariate*.

b *Adjusted for age and sex*.

c *Adjusted for age, sex, income, area of residence, Charlson comorbidity index (0, 1, 2, 3, 4, ≥ 5), glaucoma, and cataract severity*.

**Table 4:** Hazard ratios for all-cause and cause-specific mortality in elderly patients with macular diseases stratified by vitrectomy status.

**Cause of mortality**	**Unadjusted Cox model ^a^**		**Adjusted Cox model 1 ^a,b^**		**Adjusted Cox model 2 ^a,c^**	
**(No. of participants)**	**Hazard ratio (95% CI)**	***P*** **-value**	**Hazard ratio (95% CI)**	***P*** **-value**	**Hazard ratio (95% CI)**	***P*** **-value**
All-cause	0.46 (0.38–0.56)	<0.001	0.65 (0.54–0.79)	<0.001	0.64 (0.53–0.78)	<0.001
Cancer	0.62 (0.44–0.87)	<0.001	0.74 (0.53–1.04)	0.083	0.73 (0.52–1.02)	0.069
Vascular	0.48 (0.33–0.69)	<0.001	0.70 (0.48–1.01)	0.059	0.69 (0.48–1.00)	0.052
Pulmonary	0.23 (0.10–0.51)	<0.001	0.36 (0.16–0.81)	0.014	0.35 (0.16–0.79)	0.011
Neurologic	0.25 (0.06–1.00)	0.050	0.38 (0.09–1.52)	0.172	0.36 (0.09–1.46)	0.153
Infectious	0.46 (0.15–1.42)	0.174	0.64 (0.20–1.99)	0.439	0.62 (0.20–1.92)	0.403
Accident or trauma	0.94 (0.54–1.64)	0.838	1.20 (0.69–2.08)	0.525	1.21 (0.70–2.11)	0.492

*CI, confidence interval*.

a *Cox model with vitreoretinal vitrectomy status as the time-varying covariate*.

b *Model 1: adjusted for age and sex*.

c *Model 2: adjusted for age, sex, income, area of residence, Charlson comorbidity index (0, 1, 2, 3, 4, ≥ 5), glaucoma, and cataract severity*.

## Discussion

Using the NHIS database, the present study aimed to examine mortality rates according to vitrectomy status in elderly patients diagnosed with vitreoretinal diseases. The overall mortality rate of patients with retinal diseases did not differ according to vitrectomy status; however, the cause-specific mortality was different between groups. Specifically, the risk of mortality owing to pulmonary causes was significantly lower in the vitrectomy group. In addition, the adjusted all-cause and cause-specific mortality in the vitrectomy group were different according to the underlying retinal disease. A strength of this study is that it demonstrates the characteristics and patterns of mortality of patients undergoing vitrectomy and evaluates the associations between vitrectomy and socio-demographic factors.

The association between vitrectomy and mortality was modified by the effects of age in patients with vitreoretinal diseases. Patients who underwent vitrectomy were significantly younger, indicating that younger patients (≤ 70 years) often choose surgical treatment. Meanwhile, patients aged ≥ 85 years showed the lowest mortality risk associated with vitrectomy. In particular, the analysis of the vitreoretinal diseases overall were found to be associated with a decreased risk of pulmonary-related deaths. Though no studies have evaluated the association between vitrectomy and mortality from pulmonary causes, we suggest that patients who undergo vitrectomy have lower rates of mortality from pulmonary causes, possibly since their general state of health is good, meaning they can ambulate independently and receive routine medical care. The other possibility is that vision recovery after surgery is associated with an increase in physical activity, which results in fewer complications and a higher long-term survival rate. Further studies are necessary to better clarify the mechanisms underlying the association between vitrectomy and mortality from pulmonary causes.

Interestingly, the association of mortality and vitrectomy among the patients with vitreoretinal diseases differed from those of mortality and cataract or glaucoma surgery in patients with cataract or glaucoma. While mortality due to pulmonary causes was decreased following vitrectomy, according to our previous studies examining mortality after cataract surgery and glaucoma surgery (17, 18), all-cause mortality and mortality due to vascular and neurologic causes decreased in the cataract surgery group. On the other hand, all-cause mortality and, in particular, mortality from neurologic causes, increased in the glaucoma surgery group. These differences might be caused by the differences in the characteristics of the ophthalmic diseases and the indications for surgical decisions.

To analyze the differences in mortality in the vitrectomy group according to the underlying retinal disease etiology, patients were categorized into two subgroups, which accounted for more than half of all vitreoretinal diseases. When patients with retinal vascular disease and macular disease were examined separately, different associations were observed. As expected, our results showed that patients in the older adult population with retinal vascular diseases who underwent vitrectomy had a higher CCI score and systemic vascular risk and, consequently, had a higher risk of mortality from all causes and vascular causes owing to the cardiovascular risk after adjusting for demographic characteristics and systemic and ocular comorbidities. Consistent with our results, a recently published study suggested that the severity of diabetic retinopathy may provide valuable insights into patients' risk of mortality from all causes and vascular causes (19). This tendency was particularly evident in the younger patient group (aged 60–69), and it can be speculated that older adults often choose surgery when vascular risk factors are relatively well controlled.

Furthermore, we demonstrated decreased mortality in the vitrectomy group from all causes and pulmonary causes for patients with macular diseases after adjusting for demographic characteristics and systemic and ocular comorbidities. In addition, a decline in the mortality rate following vitrectomy was observed, especially in the elderly. This is a rather contradictory result, considering that a greater proportion of the patients with macular diseases who underwent vitrectomy were current or ex-smokers who regularly consumed alcohol. The main aim of surgical treatment for patients with macular disease is to facilitate better vision in most cases. Older patients who underwent vitrectomy to restore their vision may have been in good general health and thus had lower mortality rates from and vascular causes. Taken together, vitreoretinal surgeons should focus more on improving visual restoration with macular diseases.

This study has some limitations. The main limitation was the observational nature of the study. Though an association between vitrectomy and mortality was observed, it was difficult to determine the extent to which the diagnosis of retinal disease or the performance of vitrectomy contributed to the outcomes. Second, various factors, including surgeons' and patients' preferences and disease severity, must be considered when interpreting our results, however, these are unavoidable limitations to using claims data. In addition, the NHIS-Senior database does not provide information on visual acuity measurements, preoperative retinal status, or the postoperative clinical course. Therefore, we could not determine if the difference between the long-term survival rates in the vitrectomy group was directly associated with the ocular changes following vitrectomy. Third, several variables could be confirmed only for a subset of patients who underwent the national health screening program and approximately 50% of these data were missing. Fourth, we did not consider the number of surgeries in our analysis. Patients with severe diseases are more likely to need more surgeries, which may be related to mortality rates. However, since we included only the time point of the first surgery in the analysis without considering the number of surgeries and the overall course, these aspects should be considered when interpreting the study results. Lastly, we focused only on residents of South Korea; therefore, the observed findings cannot be generalized to other ethnic groups. Despite these limitations, this study is valuable. This was the first study to report a significant association between vitrectomy and long-term survival based on all-cause and cause-specific mortality in older adults using a nationwide population-based database. This study also included a large sample; the data were obtained from the NHIS-Senior database, and selection bias was relatively low because Korea has a single public insurance system that covers the entire population (20, 21).

This nationwide cohort study showed that the vitrectomy group had the lower mortality from pulmonary causes with no association for all-cause mortality. In addition, associations varied according to two types of vitreoretinal disease: higher risk of all-cause mortality and vascular causes in the patients with retinal vascular diseases and lower risk of all-cause mortality, vascular causes, and pulmonary causes in those with macular diseases. Though causality requires further study and analysis, the effect of vitrectomy on the mortality rates of patients can be inferred from the current observations. Further, the expected direction of changes of the future could be predicted based on the different mortality rate after vitrectomy in macular and retinal vascular disease.

## Data Availability Statement

Korean claims data used for this study is available only when accessed on Cloud, therefore the database set cannot be exported from the system. Further inquiries can be directed to the corresponding authors.

## Ethics Statement

The studies involving human participants were reviewed and approved by Asan Medical Center IRB. Written informed consent for participation was not required for this study in accordance with the national legislation and the institutional requirements.

## Author Contributions

YJK, HL, and JYK contributed to conception and design of the study. JSL performed the statistical analysis. YJK wrote the first draft of the manuscript. HL, JYK, and HWT wrote sections of the manuscript. All authors contributed to manuscript revision, read, and approved the submitted version.

## Funding

This work was supported by the Korea Medical Device Development Fund grant funded by the Korea government (the Ministry of Science and ICT, the Ministry of Trade, Industry and Energy, the Ministry of Health and Welfare, the Ministry of Food and Drug Safety) (Project Number: 9991006821, KMDF_PR_20200901_0148), Korean Fund for Regenerative Medicine funded by Ministry of Science and ICT, and Ministry of Health and Welfare (21C0723L1-11, Republic of Korea), a grant from the Asan Institute for Life Sciences, Asan Medical Center, Seoul, Korea (2021IL0034, 2021IP0060), and a grant (NRF-2018R1D1A1B07043010) from the National Research Foundation of Korea.

## Conflict of Interest

The authors declare that the research was conducted in the absence of any commercial or financial relationships that could be construed as a potential conflict of interest.

## Publisher's Note

All claims expressed in this article are solely those of the authors and do not necessarily represent those of their affiliated organizations, or those of the publisher, the editors and the reviewers. Any product that may be evaluated in this article, or claim that may be made by its manufacturer, is not guaranteed or endorsed by the publisher.

## References

[1] Kim MJ Park KH Hwang JM Yu HG Yu YS Chung H. The safety and efficacy of transconjunctival sutureless 23-gauge vitrectomy. Korean J Ophthalmol. (2007) 21:201–7. 10.3341/kjo.2007.21.4.201 18063883 PMC2629884

[2] Yang SJ Yoon SY Kim JG Yoon YH. Transconjunctival sutureless vitrectomy for the treatment of vitreoretinal complications in patients with diabetes mellitus. Ophthalmic Surg Lasers Imaging. (2009) 40:461–6. 10.3928/15428877-20090901-04 19772269

[3] Wubben TJ Talwar N Blachley TS Gardner TW Johnson MW Lee PP. Rates of Vitrectomy among Enrollees in a United States Managed Care Network, 2001–2012. Ophthalmology. (2016) 123:590–8. 10.1016/j.ophtha.2015.11.001 26746595

[4] Kim JY Rim TH Kim SS. Trends of Pars Plana Vitrectomy Rates in South Korea: A Nationwide Cohort Study. Korean J Ophthalmol. (2017) 31:446–51. 10.3341/kjo.2016.0070 28914000 PMC5636721

[5] Fong CS Mitchell P Rochtchina E Teber ET Hong T Wang JJ. Correction of visual impairment by cataract surgery and improved survival in older persons: the Blue Mountains Eye Study cohort. Ophthalmology. (2013) 120:1720–7. 10.1016/j.ophtha.2013.02.009 23664468

[6] Tseng VL Yu F Lum F Coleman AL. Cataract Surgery and Mortality in the United States Medicare Population. Ophthalmology. (2016) 123:1019–26. 10.1016/j.ophtha.2015.12.033 26854033

[7] Tseng VL Chlebowski RT Yu F Cauley JA Li W Thomas F Virnig BA Coleman AL. Association of cataract surgery with mortality in older women: findings from the women's health initiative. JAMA Ophthalmol. (2018) 136:3–10. 10.1001/jamaophthalmol.2017.4512 29075781 PMC5833601

[8] Liu E Estevez J Kaidonis G Hassall M Phillips R Raymond G. Long-term survival rates of patients undergoing vitrectomy for diabetic retinopathy in an Australian population: a population-based audit. Clin Exp Ophthalmol. (2019) 47:598–604. 10.1111/ceo.13466 30663192

[9] Banerjee PJ Moya R Bunce C Charteris DG Yorston D Wickham L. Long-term survival rates of patients undergoing vitrectomy for proliferative diabetic retinopathy. Ophthalmic Epidemiol. (2016) 23:94–8. 10.3109/09286586.2015.1089578 26954846

[10] Shukla SY Hariprasad AS Hariprasad SM. Long-term mortality in diabetic patients with tractional retinal detachments. Ophthalmol Retina. (2017) 1:8–11. 10.1016/j.oret.2016.09.002 31047400

[11] Kim YI Kim YY Yoon JL Won CW Ha S Cho KD. Cohort profile: national health insurance service-senior (NHIS-senior) cohort in Korea. BMJ Open. (2019) 9:e024344. 10.1136/bmjopen-2018-024344 31289051 PMC6615810

[12] Ryu SY Kim J Hong JH Chung EJ. Incidence and characteristics of cataract surgery in South Korea from 2011 to 2015: a nationwide population-based study. Clin Exp Ophthalmol. (2020) 48:319–27. 10.1111/ceo.13705 31867796

[13] Levesque LE Hanley JA Kezouh A Suissa S. Problem of immortal time bias in cohort studies: example using statins for preventing progression of diabetes. BMJ. (2010) 340:b5087. 10.1136/bmj.b5087 20228141

[14] Charlson ME Pompei P Ales KL MacKenzie CR. A new method of classifying prognostic comorbidity in longitudinal studies: development and validation. J Chronic Dis. (1987) 40:373–83. 10.1016/0021-9681(87)90171-8 3558716

[15] Austin PC. Balance diagnostics for comparing the distribution of baseline covariates between treatment groups in propensity-score matched samples. Stat Med. (2009) 28:3083–107. 10.1002/sim.3697 19757444 PMC3472075

[16] Mamdani M Sykora K Li P Normand SL Streiner DL Austin PC. Reader's guide to critical appraisal of cohort studies: 2. Assessing potential for confounding. BMJ. (2005) 330:960–2. 10.1136/bmj.330.7497.960 15845982 PMC556348

[17] Kim JY Chung HS Lee JS Lee H Tchah H. Relationship between cataract surgery and mortality in elderly patients with cataract: nationwide population-based cohort study in South Korea. J Pers Med. (2021) 11:1128. 10.3390/jpm11111128 34834480 PMC8625327

[18] Lee SY Lee H Lee JS Han SA Kim YJ Kim JY. Association between glaucoma surgery and all-cause and cause-specific mortality among elderly patients with glaucoma: a nationwide population-based cohort study. Sci Rep. (2021) 11:17055. 10.1038/s41598-021-96063-7 34426612 PMC8382742

[19] Modjtahedi BS Wu J Luong TQ Gandhi NK Fong DS Chen W. Severity of diabetic retinopathy and the risk of future cerebrovascular disease, cardiovascular disease, all-cause mortality. Ophthalmology. (2020) 128:1169–79. 10.1016/j.ophtha.2020.12.019 33359888

[20] Seong SC Kim YY Park SK Khang YH Kim HC Park JH. Cohort profile: the National Health Insurance Service-National Health Screening Cohort (NHIS-HEALS) in Korea. BMJ Open. (2017) 7:e016640. 10.1136/bmjopen-2017-016640 28947447 PMC5623538

[21] Lee J Lee JS Park SH Shin SA Kim K. Cohort Profile: The National Health Insurance Service-National Sample Cohort (NHIS-NSC), South Korea. Int J Epidemiol. (2017) 46:e15. 10.1093/ije/dyv319 26822938

