# Supplementary material for: Vitrectomy and All-Cause and Cause-Specific Mortality in Elderly Patients With Vitreoretinal Diseases: A Nationwide Cohort Study
Source: Front Med (Lausanne). 2022 Apr 25;9:851536. doi: 10.3389/fmed.2022.851536 (PMC9082070; doi:10.3389/fmed.2022.851536)
Supplement: Supplementary file 1 [file Data_Sheet_1.docx]

**Supplementary Table 1**. Korean Standard Classification of Diseases and Korean Electronic Data Interchange codes for retinal diseases

| **Inclusion criteria** | **Eligible KCD Diagnostic Codes** |
| --- | --- |
| Chorioretinal inflammation | H30.0, H30.1, H30.2, H30.8, H 30.9, H 32.0 |
| Chorioretinal scar/degeneration | H31.0, H31.1 |
| Choroidal hemorrhage/rupture | H31.3, H31.4 |
| Degeneration of the macula and posterior pole | H35.30–H35.39 |
| Degenerative myopia | H44.2 |
| Degeneration of the peripheral retina | H35.2, H35.40, H35.41, H35.42, H35.43, H35.44, H35.45, H35.49, H35.70, H35.71, H35.72, H35.79 |
| Diabetic retinopathy | H35.0,36.0, E10.3, E11.3, E12.3, E13.3 E14.3 |
| Endophthalmitis | H45.1, H44.0, H44.1 |
| Hemorrhage of the retina and vitreous | H35.6, H43.1, H45.0 |
| Intraocular foreign body | H44.6, H44.7 |
| Retinal artery occlusion | H34.0, H34.1, H34.2 |
| Retinal detachment/retinal break | H31.4, H33.00, H 33.01, H,33.02, H 33.03, H33.04, H33.09 H33.10, H33.18, H33.19, H33.30, H33.31, H33.39, H33.4, H33.50, H33.58 |
| Retinal vein occlusion | H34.8, H34.9 |
| Vitreous opacities | H43.8, H43.2, H43.30, H43.38, H43.80, H43.88, H43.8 |
| **Exposure** | **Eligible KEDI Code** |
| Vitrectomy | S5121 |

Abbreviations: KCD, Korean Standard Classification of Diseases; KEDI, Korean Electronic Data Interchange.

**Supplementary Table 2**. Korean Standard Classification of Diseases codes for mortality attributed to specific systemic conditions

| **Type of Mortality** | **Eligible KCD Diagnostic Codes** |
| --- | --- |
| Cancer death | C00–C14, C15–C26, C30–C39, C40–C41, C43–C44, C45–C49. C50, C51–C58, C60–C63, C64–C68, C69–C72, C73–C75, C76–C80, C81–C96, C97–C97, D00–D09, D10–D36, D37–D48 |
| Vascular death | I00–I02, I05–I09, I10–I15, I20–I25, I26–I28, I30–I52, I60–I69, I70–I79, I80–I89, I95–I99 |
| Pulmonary death | J00–J06, J09–J18, J20–J22, J30–J39, J40–J47, J60–J70, J80–J84, J85–J86, J90–J94, J95–J99 |
| Neurologic death | G00–G09, G10–G14, G20–G26, G30–G32, G35–G37, G40–G47, G50–G59, G60–G64, G70–G73, G80–G83, G90–G99 |
| Infection-related death | A00–A09, A15–A19, A20–A28, A30–A49, A50–A64, A65–A69, A70–A74, A75–A79, A80–A89, A90–A99, B00–B09, B15–B19, B20–B24, B25–B34, B35–B49, B50–B64, B65–B83, B85–B89, B90–B94, B95–B98, B99 |
| Accident or trauma-related death | V01–V99, W00–W99, X00–X84, X85–Y09, Y10–Y34, Y35–Y36, Y40–Y59, Y60–Y69, Y70–Y82, Y83–Y84, Y85–Y89, Y90–Y98 |

Abbreviations: KCD, Korean Standard Classification of Diseases.

**Supplementary Table 3**. Korean Standard Classification of Diseases codes for comorbidities

| **Comorbidity** | **Eligible KCD Diagnostic Codes** |
| --- | --- |
| Lymphomas | C81–C86, C88 |
| Multiple myeloma/leukemia | C90–C96 |
| Malignant neoplasms | C00–97 |
| Myocardial infarction | I21–I23 |
| Heart failure | I50, I97.1, I09.9, I11.0, I13.0, I13.2 |
| Cerebrovascular disease | I60–I69 |
| Peripheral vascular disease | I73 |
| Chronic pulmonary disease | J44, I27.9, J84 |
| Cirrhosis | K70.3, K71.7, K74, K76.1 |
| Hepatic failure | K70.4, K71.1, K72 |
| AIDS | B20–B24 |
| Hemi/paraplegia | G04.1, G11.4, G81–82, I69.006, I69.106, I69.206, I69.306, I69.406 |
| Rheumatologic disease | M05, M06, M10, M12.0, M12.3, M30–M36 |
| Dementia | F00–F03, F05.1, G31.82 |
| Diabetes mellitus | E10–E14 |
| Diabetes mellitus with complications | E10.0–E10.8, E11.0–E11.8, E12.0–E12.8, E13.0–E13.8, E14.0–E14.8 |
| Chronic renal disease | E10.22, E12.22, E13.22, E14.22, I12, I13 |
| Peptic ulcer disease | K27 |
| Glaucoma | H40, H42 |
| Age-related macular degeneration | H35.30, H35.31, H35.39 |
| Diabetes mellitus with ophthalmic manifestations | E10.3^a^, E11.3^a^, E12.3^a^, E13.3^a^, E14.3^a^ |

Abbreviations: KCD, Korean Standard Classification of Diseases; AIDS, acquired immune deficiency syndrome.

^a^1, 2, 3, 4, 8.

**Supplementary Table 4**. Demographic and baseline characteristics of the subjects according to vitrectomy status

| Variable | Total  (N=152,283) | Vitrectomy group (n=3,313) | Non-vitrectomy group (n=148,970) | ASD^a^ |
| --- | --- | --- | --- | --- |
| Age (years) |  |  |  | 0.4047 |
| < 70 | 55,108 (36.2) | 1,691 (51.0) | 53,417 (35.9) |  |
| 70–74 | 47,304 (31.1) | 1,025 (30.9) | 46,279 (31.1) |  |
| 75–79 | 29,675 (19.5) | 428 (12.9) | 29,247 (19.6) |  |
| 80–84 | 14,206 (9.3) | 131 (4.0) | 14,075 (9.4) |  |
| ≥ 85 | 5,990 (3.9) | 38 (1.1) | 5,952 (4.0) |  |
| Mean ± SD | 72.3 ± 6.2 | 69.9 ± 5.3 | 72.4 ± 6.2 | 0.4245 |
| Sex |  |  |  | 0.0667 |
| Male | 57,342 (37.7) | 1,353 (40.8) | 55,989 (37.6) |  |
| Female | 94,941 (62.3) | 1,960 (59.2) | 92,981 (62.4) |  |
| Area of residence |  |  |  | 0.1085 |
| Metropolitan | 63,642 (41.8) | 1,559 (47.1) | 62,083 (41.7) |  |
| Provincial | 88,641 (58.2) | 1,754 (52.9) | 86,887 (58.3) |  |
| Income |  |  |  | 0.1214 |
| Below 20^th^ percentile | 36,625 (24.1) | 635 (19.2) | 35,990 (24.2) |  |
| Above 20^th^ percentile | 115,658 (75.9) | 2,678 (80.8) | 112,980 (75.8) |  |
| CCI |  |  |  | 0.0665 |
| 0 | 18,766 (12.3) | 416 (12.6) | 18,350 (12.3) |  |
| 1 | 25,861 (17.0) | 615 (18.6) | 25,246 (16.9) |  |
| 2 | 26,541 (17.4) | 565 (17.1) | 25,976 (17.4) |  |
| 3 | 22,911 (15.0) | 521 (15.7) | 22,390 (15.0) |  |
| 4 | 18,314 (12.0) | 408 (12.3) | 17,906 (12.0) |  |
| ≥ 5 | 39,890 (26.2) | 788 (23.8) | 39,102 (26.2) |  |
| Smoking status, n (%) |  |  |  | 0.0191 |
| Non-smoker | 66,316 (43.5) | 1,418 (42.8) | 64,898 (43.6) |  |
| Ex-smoker | 7,734 (5.1) | 165 (5.0) | 7,569 (5.1) |  |
| Current smoker | 9,501 (6.2) | 204 (6.2) | 9,297 (6.2) |  |
| Missing | 68,732 (45.1) | 1,526 (46.1) | 67,206 (45.1) |  |
| Alcohol consumption, n (%) |  |  |  | 0.0313 |
| No | 65,197 (42.8) | 1,373 (41.4) | 63,824 (42.8) |  |
| Yes | 19,044 (12.5) | 438 (13.2) | 18,606 (12.5) |  |
| Missing | 68,042 (44.7) | 1,502 (45.3) | 66,540 (44.7) |  |
| Regular exercise, n (%) |  |  |  | 0.0111 |
| No | 70,993 (46.6) | 1,534 (46.3) | 69,459 (46.6) |  |
| Yes | 14,122 (9.3) | 301 (9.1) | 13,821 (9.3) |  |
| Missing | 67,168 (44.1) | 1,478 (44.6) | 65,690 (44.1) |  |
| Glaucoma |  |  |  | 0.2203 |
| No | 93,321 (61.3) | 1,678 (50.6) | 91,643 (61.5) |  |
| Yes | 58,962 (38.7) | 1,635 (49.4) | 57,327 (38.5) |  |

Abbreviations: ASD, absolute standardized difference; CCI, Charlson comorbidity Index; SD, standard deviation.Data are expressed as mean ± SD or n (%).

^a^ASD > 0.1 is considered a meaningful imbalance.

**Supplementary Table 5**. Hazard ratios for all-cause mortality in elderly patients with vitreoretinal diseases stratified by age, sex, area of residence, income, Charlson comorbidity index score, and ocular comorbidities

|  | Adjusted hazard ratio (95% CI) | *P*-value | *P*-value for interaction |
| --- | --- | --- | --- |
| Age (years) |  |  | <0.001 |
| < 70 | 1.21 (1.07–1.37) | 0.003 |  |
| 70–74 | 0.88 (0.75–1.04) | 0.140 |  |
| 75–79 | 0.81 (0.66–1.00) | 0.052 |  |
| 80–84 | 0.61 (0.45–0.84) | 0.003 |  |
| ≥ 85 | 0.44 (0.25–0.78) | 0.005 |  |
| Sex |  |  | 0.129 |
| Male | 1.02 (0.90–1.15) | 0.760 |  |
| Female | 0.89 (0.78–1.01) | 0.075 |  |
| Area of residence |  |  | 0.669 |
| Metropolitan | 0.98 (0.86–1.11) | 0.724 |  |
| Provincial | 0.94 (0.83–1.06) | 0.317 |  |
| Income |  |  | 0.824 |
| Below 20^th^ percentile | 0.94 (0.78–1.13) | 0.507 |  |
| Above 20^th^ percentile | 0.96 (0.87–1.06) | 0.448 |  |
| CCI |  |  | 0.521 |
| 0 | 0.97 (0.75–1.27) | 0.844 |  |
| 1 | 0.81 (0.63–1.04) | 0.093 |  |
| 2 | 0.95 (0.75–1.20) | 0.647 |  |
| 3 | 1.14 (0.91–1.41) | 0.259 |  |
| 4 | 0.92 (0.72–1.19) | 0.537 |  |
| ≥ 5 | 0.96 (0.83–1.11) | 0.575 |  |
| Severe cataract |  |  | 0.057 |
| No | 0.92 (0.83–1.02) | 0.0990 |  |
| Yes | 1.13 (0.94–1.35) | 0.2034 |  |
| Glaucoma |  |  | 0.969 |
| No | 0.96 (0.84–1.08) | 0.471 |  |
| Yes | 0.96 (0.85–1.08) | 0.502 |  |

Abbreviations: CCI, Charlson comorbidity index; CI, confidence interval.

The non-vitrectomy group was used as the reference for all models.

Adjusted for age, sex, income, area of residence, Charlson comorbidity index (0, 1, 2, 3, 4, ≥ 5), glaucoma, and cataract severity.

**Supplementary Table 6**. Demographic and baseline characteristics of patients with retinal vascular diseases based on vitrectomy status

| Variable | Total  (N=57,825) | Vitrectomy group (n=1,203) | Non-vitrectomy group (n=56,622) | ASD^a^ |
| --- | --- | --- | --- | --- |
| Age (years) |  |  |  | 0.5152 |
| < 70 | 21752 (37.6) | 692 (57.5) | 21060 (37.2) |  |
| 70–74 | 18126 (31.3) | 353 (29.3) | 17773 (31.4) |  |
| 75–79 | 11042 (19.1) | 115 (9.6) | 10927 (19.3) |  |
| 80–84 | 5043 (8.7) | 35 (2.9) | 5008 (8.8) |  |
| ≥ 85 | 1862 (3.2) | 8 (0.7) | 1854 (3.3) |  |
| Mean ± SD | 72.0 ± 6.0 | 69.0 ± 5.0 | 72.1 ± 6.0 | 0.5482 |
| Sex |  |  |  | 0.1033 |
| Male | 22834 (39.5) | 535 (44.5) | 22299 (39.4) |  |
| Female | 34991 (60.5) | 668 (55.5) | 34323 (60.6) |  |
| Area of residence |  |  |  | 0.1347 |
| Metropolitan | 24996 (43.2) | 599 (49.8) | 24397 (43.1) |  |
| Provincial | 32829 (56.8) | 604 (50.2) | 32225 (56.9) |  |
| Income |  |  |  | 0.0742 |
| Below 20^th^ percentile | 14067 (24.3) | 256 (21.3) | 13811 (24.4) |  |
| Above 20^th^ percentile | 43758 (75.7) | 947 (78.7) | 42811 (75.6) |  |
| CCI |  |  |  | 0.1377 |
| 0 | 3947 (6.8) | 93 (7.7) | 3854 (6.8) |  |
| 1 | 6375 (11.0) | 150 (12.5) | 6225 (11.0) |  |
| 2 | 7719 (13.3) | 143 (11.9) | 7576 (13.4) |  |
| 3 | 8534 (14.8) | 206 (17.1) | 8328 (14.7) |  |
| 4 | 8270 (14.3) | 196 (16.3) | 8074 (14.3) |  |
| ≥ 5 | 22980 (39.7) | 415 (34.5) | 22565 (39.9) |  |
| Smoking status, n (%) |  |  |  | 0.1161 |
| Non-smoker | 23548 (40.7) | 442 (36.7) | 23106 (40.8) |  |
| Ex-smoker | 2933 (5.1) | 49 (4.1) | 2884 (5.1) |  |
| Current smoker | 3501 (6.1) | 66 (5.5) | 3435 (6.1) |  |
| Missing | 27843 (48.2) | 646 (53.7) | 27197 (48.0) |  |
| Alcohol consumption, n (%) |  |  |  | 0.1177 |
| No | 23498 (40.6) | 433 (36.0) | 23065 (40.7) |  |
| Yes | 6742 (11.7) | 127 (10.6) | 6615 (11.7) |  |
| Missing | 27585 (47.7) | 643 (53.4) | 26942 (47.6) |  |
| Regular exercise, n (%) |  |  |  | 0.1205 |
| No | 25168 (43.5) | 465 (38.7) | 24703 (43.6) |  |
| Yes | 5396 (9.3) | 100 (8.3) | 5296 (9.4) |  |
| Missing | 27261 (47.1) | 638 (53.0) | 26623 (47.0) |  |
| Severe cataract |  |  |  | 0.0384 |
| No | 49331 (85.3) | 1010 (84.0) | 48321 (85.3) |  |
| Yes | 8494 (14.7) | 193 (16.0) | 8301 (14.7) |  |
| Glaucoma |  |  |  | 0.2088 |
| No | 36886 (63.8) | 647 (53.8) | 36239 (64.0) |  |
| Yes | 20939 (36.2) | 556 (46.2) | 20383 (36.0) |  |

Abbreviations: ASD, absolute standardized difference; CCI, Charlson comorbidity Index; SD, standard deviation.

Data are expressed as mean ± SD or n (%).

^a^ASD > 0.1 is considered a meaningful imbalance.

**Supplementary Table 7**. Demographic and baseline characteristics of patients with macular diseases based on vitrectomy status

| Variable | Total  (N=33,915) | Vitrectomy group (n=1,002) | Non-vitrectomy group (n=32,913) | ASD^a^ |
| --- | --- | --- | --- | --- |
| Age (years) |  |  |  | 0.5547 |
| < 70 | 9503 (28.0) | 463 (46.2) | 9040 (27.5) |  |
| 70–74 | 10485 (30.9) | 338 (33.7) | 10147 (30.8) |  |
| 75–79 | 7774 (22.9) | 146 (14.6) | 7628 (23.2) |  |
| 80–84 | 4212 (12.4) | 46 (4.6) | 4166 (12.7) |  |
| ≥ 85 | 1941 (5.7) | 9 (0.9) | 1932 (5.9) |  |
| Mean ± SD | 73.6 ± 6.3 | 70.5 ± 5.0 | 73.7 ± 6.3 | 0.5668 |
| Sex |  |  |  | 0.0181 |
| Male | 12301 (36.3) | 355 (35.4) | 11946 (36.3) |  |
| Female | 21614 (63.7) | 647 (64.6) | 20967 (63.7) |  |
| Area of residence |  |  |  | 0.1148 |
| Metropolitan | 13568 (40.0) | 456 (45.5) | 13112 (39.8) |  |
| Provincial | 20347 (60.0) | 546 (54.5) | 19801 (60.2) |  |
| Income |  |  |  | 0.1773 |
| Below 20^th^ percentile | 8636 (25.5) | 184 (18.4) | 8452 (25.7) |  |
| Above 20^th^ percentile | 25279 (74.5) | 818 (81.6) | 24461 (74.3) |  |
| CCI |  |  |  | 0.0637 |
| 0 | 5012 (14.8) | 148 (14.8) | 4864 (14.8) |  |
| 1 | 6812 (20.1) | 213 (21.3) | 6599 (20.0) |  |
| 2 | 6675 (19.7) | 204 (20.4) | 6471 (19.7) |  |
| 3 | 5143 (15.2) | 158 (15.8) | 4985 (15.1) |  |
| 4 | 3672 (10.8) | 106 (10.6) | 3566 (10.8) |  |
| ≥ 5 | 6601 (19.5) | 173 (17.3) | 6428 (19.5) |  |
| Smoking status, n (%) |  |  |  | 0.1587 |
| Non-smoker | 14543 (42.9) | 483 (48.2) | 14060 (42.7) |  |
| Ex-smoker | 1646 (4.9) | 59 (5.9) | 1587 (4.8) |  |
| Current smoker | 2110 (6.2) | 74 (7.4) | 2036 (6.2) |  |
| Missing | 15616 (46.0) | 386 (38.5) | 15230 (46.3) |  |
| Alcohol consumption, n (%) |  |  |  | 0.1726 |
| No | 14330 (42.3) | 479 (47.8) | 13851 (42.1) |  |
| Yes | 4128 (12.2) | 148 (14.8) | 3980 (12.1) |  |
| Missing | 15457 (45.6) | 375 (37.4) | 15082 (45.8) |  |
| Regular exercise, n (%) |  |  |  | 0.1805 |
| No | 15744 (46.4) | 525 (52.4) | 15219 (46.2) |  |
| Yes | 2903 (8.6) | 110 (11.0) | 2793 (8.5) |  |
| Missing | 15268 (45.0) | 367 (36.6) | 14901 (45.3) |  |
| Severe cataract |  |  |  | 0.0215 |
| No | 26895 (79.3) | 803 (80.1) | 26092 (79.3) |  |
| Yes | 7020 (20.7) | 199 (19.9) | 6821 (20.7) |  |
| Glaucoma |  |  |  | 0.1653 |
| No | 19960 (58.9) | 510 (50.9) | 19450 (59.1) |  |
| Yes | 13955 (41.1) | 492 (49.1) | 13463 (40.9) |  |

Abbreviations: ASD, absolute standardized difference; CCI, Charlson comorbidity Index; SD, standard deviation.

Data are expressed as mean ± SD or n (%).

^a^ASD > 0.1 is considered a meaningful imbalance.

**Supplementary Table 8**. Hazard ratios for all-cause mortality in elderly patients with retinal vascular diseases stratified by age, sex, area of residence, income, Charlson comorbidity index score, and ocular comorbidities

|  | Adjusted hazard ratio (95% CI) | *P*-value | *P*-value for interaction |
| --- | --- | --- | --- |
| Age (years) |  |  | 0.0507 |
| < 70 | 1.46 (1.24–1.71) | <.0001 |  |
| 70–74 | 0.98 (0.77–1.26) | 0.8960 |  |
| 75–79 | 1.17 (0.88–1.57) | 0.2806 |  |
| 80–84 | 0.86 (0.52–1.42) | 0.5586 |  |
| ≥ 85 | 1.37 (0.45–4.13) | 0.5816 |  |
| Sex |  |  | 0.5229 |
| Male | 1.30 (1.10–1.53) | 0.0016 |  |
| Female | 1.20 (1.00–1.44) | 0.0457 |  |
| Area of residence |  |  | 0.8435 |
| Metropolitan | 1.27 (1.07–1.51) | 0.0068 |  |
| Provincial | 1.24 (1.05–1.47) | 0.0113 |  |
| Income |  |  | 0.736 |
| Below 20^th^ percentile | 1.21 (0.94–1.55) | 0.1341 |  |
| Above 20^th^ percentile | 1.27 (1.11–1.46) | 0.0007 |  |
| CCI |  |  | 0.0665 |
| 0 | 1.16 (0.73–1.84) | 0.5367 |  |
| 1 | 1.02 (0.67–1.55) | 0.9234 |  |
| 2 | 1.62 (1.16–2.27) | 0.0051 |  |
| 3 | 1.75 (1.34–2.29) | <.0001 |  |
| 4 | 1.10 (0.80–1.50) | 0.5617 |  |
| ≥ 5 | 1.17 (0.97–1.41) | 0.1101 |  |
| Severe cataract |  |  | 0.0244 |
| No | 1.18 (1.03–1.35) | 0.0166 |  |
| Yes | 1.65 (1.27–2.14) | 0.0001 |  |
| Glaucoma |  |  | 0.9775 |
| No | 1.25 (1.06–1.48) | 0.0073 |  |
| Yes | 1.26 (1.05–1.50) | 0.0108 |  |

Abbreviations: CCI, Charlson comorbidity index; CI, confidence interval.

The non-vitrectomy group was used as the reference for all models.

Adjusted for age, sex, income, area of residence, Charlson comorbidity Index (0, 1, 2, 3, 4, ≥ 5), glaucoma, and cataract severity.

**Supplementary Table 9**. Hazard ratios for all-cause mortality in elderly patients with macular diseases stratified by age, sex, area of residence, income, Charlson comorbidity index score, and ocular comorbidities

|  | Adjusted hazard ratio (95% CI) | *P*-value | *P*-value for interaction |
| --- | --- | --- | --- |
| Age (years) |  |  | 0.0862 |
| < 70 | 0.86 (0.64–1.15) | 0.3159 |  |
| 70–74 | 0.63 (0.44–0.90) | 0.0122 |  |
| 75–79 | 0.42 (0.25–0.70) | 0.0011 |  |
| 80–84 | 0.50 (0.28–0.89) | 0.0186 |  |
| ≥ 85 | 0.43 (0.19–0.94) | 0.0345 |  |
| Sex |  |  | 0.1433 |
| Male | 0.75 (0.57–0.98) | 0.0381 |  |
| Female | 0.56 (0.42–0.74) | <.0001 |  |
| Area of residence |  |  | 0.6601 |
| Metropolitan | 0.68 (0.51–0.90) | 0.0069 |  |
| Provincial | 0.62 (0.48–0.81) | 0.0004 |  |
| Income |  |  | 0.7571 |
| Below 20^th^ percentiles | 0.68 (0.46–1.00) | 0.0499 |  |
| Above 20^th^ percentiles | 0.63 (0.51–0.79) | <.0001 |  |
| CCI |  |  | 0.2062 |
| 0 | 1.08 (0.71–1.63) | 0.7179 |  |
| 1 | 0.55 (0.33–0.93) | 0.0246 |  |
| 2 | 0.55 (0.33–0.93) | 0.0250 |  |
| 3 | 0.51 (0.28–0.93) | 0.0268 |  |
| 4 | 0.57 (0.30–1.09) | 0.0912 |  |
| ≥ 5 | 0.67 (0.48–0.94) | 0.0205 |  |
| Severe cataract |  |  | 0.3999 |
| No | 0.62 (0.49–0.77) | <.0001 |  |
| Yes | 0.75 (0.51–1.10) | 0.1378 |  |
| Glaucoma |  |  | 0.7305 |
| No | 0.67 (0.51–0.87) | 0.0033 |  |
| Yes | 0.62 (0.47–0.82) | 0.0008 |  |

Abbreviations: CCI, Charlson comorbidity index; CI, confidence interval.

The non-vitrectomy group was used as the reference for all models.

Adjusted for age, sex, income, area of residence, Charlson comorbidity index (0, 1, 2, 3, 4, ≥ 5), glaucoma, and cataract severity.
